# Towards a Parsimonious Pathway Model of Modifiable and Mediating Risk Factors Leading to Diabetes Risk

**DOI:** 10.3390/ijerph182010907

**Published:** 2021-10-17

**Authors:** Yi-Ching Lynn Ho, Vivian Shu Yi Lee, Moon-Ho Ringo Ho, Gladis Jing Lin, Julian Thumboo

**Affiliations:** 1 Office of Regional Health, Singapore Health Services, 167 Jalan Bukit Merah, Singapore 150167, Singapore; vivian.lee.s.y@singhealth.com.sg (V.S.Y.L.); gladis.lin.jing@singhealth.com.sg (G.J.L.); julian.thumboo@singhealth.com.sg (J.T.); 2 Programme in Health Services and Systems Research, Duke-NUS Medical School, 8 College Rd., Singapore 169857, Singapore; 3 School of Social Sciences, Nanyang Technological University, 48 Nanyang Ave., Singapore 639818, Singapore; HOmh@ntu.edu.sg; 4 Department of Rheumatology and Immunology, Singapore General Hospital, Outram Rd., Singapore 169608, Singapore; 5 Medicine Academic Clinical Programme, Duke-NUS Medical School, 8 College Rd., Singapore 169857, Singapore

**Keywords:** diabetes, risk factors, modifiable, mediators, lifestyle, physiological, pathways, mediation analysis, structural equation modeling, Indonesia

## Abstract

Modifiable risk factors are of interest for chronic disease prevention. Few studies have assessed the system of modifiable and mediating pathways leading to diabetes mellitus. We aimed to develop a pathway model for Diabetes Risk with modifiable Lifestyle Risk factors as the start point and Physiological Load as the mediator. As there are no standardised risk thresholds for lifestyle behaviour, we derived a weighted composite for Lifestyle Risk. Physiological Load was based on an index using clinical thresholds. Sociodemographics are non-modifiable risk factors and were specified as covariates. We used structural equation modeling to test the model, first using 2014/2015 data from the Indonesian Family Life Survey. Next, we fitted a smaller model with longitudinal data (2007/2008 to 2014/2015), given limited earlier data. Both models showed the indirect effects of Lifestyle Risk on Diabetes Risk via the mediator of Physiological Load, whereas the direct effect was only supported in the cross-sectional analysis. Specifying Lifestyle Risk as an observable, composite variable incorporates the cumulative effect of risk behaviour and differentiates this study from previous studies assessing it as a latent construct. The parsimonious model groups the multifarious risk factors and illustrates modifiable pathways that could be applied in chronic disease prevention efforts.

## 1. Introduction

Diabetes mellitus alone contributed to 1.6 million deaths worldwide in 2017, a figure that is estimated to double by 2040 [1]. In terms of global burden, China (89.5 million), India (67.8 million), the USA (30.7 million), Indonesia (21.0 million), and Mexico (13.1 million) have been identified as five countries with the most diabetes cases, together with the most deaths and the highest disability-adjusted life years (DALYs) due to diabetes [2]. Among low- and middle-income countries, the disease is growing rapidly with Type 2 diabetes accounting for the majority of the cases. Three-quarters of diabetics are living in these countries [3,4].

Public health efforts in prevention and early control are needed to stem the rise in prevalence, given the disease burden from the higher risks of developing health complications, disability, and premature death [2]. Consequently, the healthcare and economic costs are also set to grow [1,2,3]. Identifying the risk factors and understanding their pathways towards disease development is thus crucial in aiding preventative and early intervention efforts [3,4,5].

The development of Type 2 diabetes has been closely associated with multiple risk factors, from demographic factors e.g., age, sex, ethnicity, socioeconomic status, and educational level, to physiological factors and behaviour, e.g., hypertension, hyperlipidaemia, high body mass index (BMI), little sleep, pulse rate, c-reactive protein, unhealthy diets, physical inactivity, and tobacco use [4,6,7,8,9,10,11,12,13,14,15]. The risk factors may be classified according to whether they are modifiable, a good example being behaviour, or non-modifiable, such as demographic and genetic factors [11]. These risk factors can also be further differentiated in terms of their temporality: From the start point of a normal baseline, indicators of unfavourable metabolic and physiological changes, such as weight gain and increased blood pressure [11], can be regarded as downstream factors caused by preceding unhealthy lifestyle-related behaviour (e.g., a lack of physical activity, poor diet, smoking and lack of sleep) [16]. Obesity is known as the leading risk factor for Type 2 diabetes [17], but in order to prevent obesity itself, we need to go upstream to the risk factors for high BMI. Indeed physiological risk factors are often associated with lifestyle behavioural factors [18,19,20]. As a risk factor for diabetes, high BMI is associated with an unhealthy diet that includes high consumption of sodium and saturated fats [3,21,22].

As predictors of diabetes, these risk factors have often been investigated in terms of their direct relationship with diabetes outcomes [23] without distinguishing between the types of risk factors and potential dependence effects in a pathway system [16]; for example, how the risk factors may influence one other (both as independent and dependent variables) to impact diabetes risk. Furthermore, it is common to use categorical outcomes of diabetes status based on diagnostic thresholds, which neglects the continuum of diabetes risk and may also result in unbalanced samples of groups with and without diabetes [24,25]. This poses a problem for statistical analyses and requires compensating techniques (e.g., oversampling) [24].

**Figure 1:**
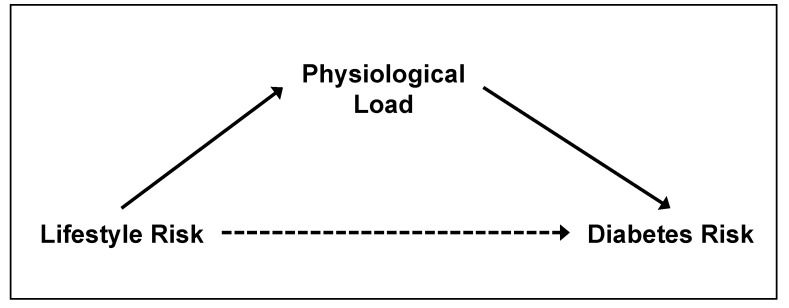
Illustration of the hypothesized pathways between Lifestyle Risk, Physiological Load and Diabetes Risk. Lifestyle Risk has a positive but indirect effect on Diabetes Risk through the mediator of Physiological Load. Note: Straight line with one arrowhead denotes positive and direct effect; dashed line with one arrowhead denotes positive but indirect effect.

To address the challenges of multiple risk factors of different types and temporal effects, we used the approach to group risk factors in terms of their hypothesised effects, thus aiming to develop a parsimonious pathway model for diabetes risk, i.e., a simple model with minimal variables yet flexible enough to incorporate various classes of risk factors to explain how diabetes risk may be modulated by health behaviour changes. It is with this in mind that the modifiable lifestyle risk factors were set as the start point of the pathways (Figure 1). Lifestyle Risk variables form a natural grouping of independent variables concerned with health-related behaviour, such as the level of physical inactivity, smoking, the consumption frequency of unhealthy food, and insufficient sleep [4,6,8,9,10]. The second group of variables is termed Physiological Load, comprising clinical monitoring indicators associated with diabetes, such as body mass index (BMI), resting pulse rate (RPR), c-reactive protein (CRP), systolic (SBP), and diastolic (DBP) blood pressure [11,16,18,19,20]. Risk factors that cannot be modified, such as sociodemographics, will be specified as covariates of each main factor, rather than as main factors themselves. This strategy allows for the necessary accounting of the effects of the non-modifiable risk factors while focusing on the impact of the modifiable factors on diabetes risk. Lastly, we used the continuous variable of HbA1c as a marker of diabetes risk, which has the advantages of avoiding unbalanced diabetes outcome categories, gaining statistical power, and allowing a more informative interpretation of the relationships.

**Figure 2:**
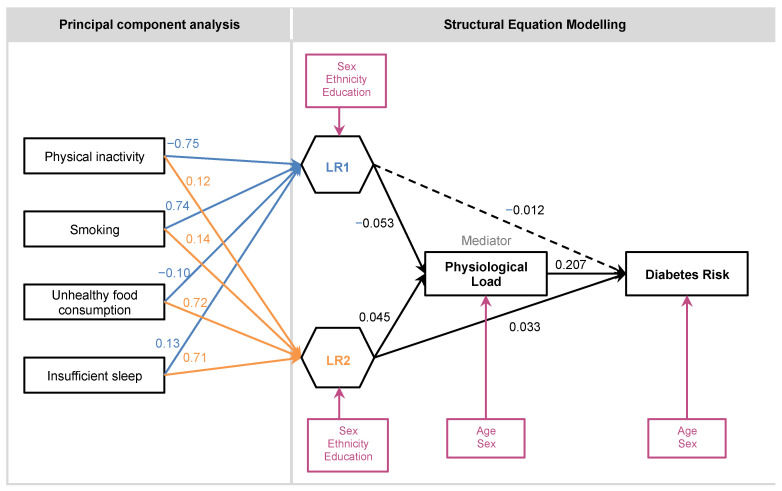
Model A: Factor loadings of the structural equation model (SEM) and the two principal components of Lifestyle Risk (LR1, LR2). Physiological Load was demonstrated to be a mediator of Lifestyle Risk to Diabetes Risk. There was also a significant direct path from Lifestyle Risk (LR2) to Diabetes Risk. Only statistically significant sociodemographic covariates (pink boxes) were included in the figure. Note: A straight line with one arrowhead denotes a significant effect; a dashed line with one arrowhead denotes an insignificant effect. Boxes indicate observed variables; hexagons indicate composite variables. Principal Component Aanalysis (PCA) loadings were rounded to 2 decimal places; Pathway load-ings in SEM model were rounded to 3 decimal places.

**Figure 3:**
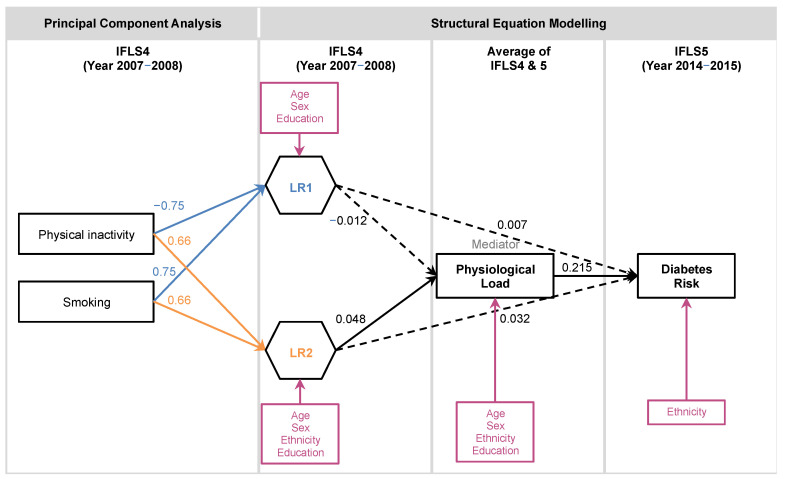
Model B: Factor loadings of the structural equation model (SEM) and the two principal components of Lifestyle Risk (LR1, LR2). Physiological Load was demonstrated to be a mediator of Lifestyle Risk (LR2) to Diabetes Risk. Only statistically significant sociodemographic covariates (pink boxes) were included in the figure. Note: A straight line with one arrowhead denotes a significant effect; a dashed line with one arrowhead denotes an insignificant effect. Boxes indicate observed variables; hexagons indicate composite variables. Principal Component Aanalysis (PCA) loadings were rounded to 2 decimal places; Pathway loadings in SEM model were rounded to 3 decimal places.

We aimed to examine the direct and indirect effects of two groups of modifiable risk factors on Diabetes Risk, namely “Lifestyle Risk” and “Physiological Load”. We hypothesised that Lifestyle Risk would have a positive but indirect effect on Diabetes Risk through the mediator of Physiological Load (Figure 1), as Lifestyle Risk is postulated to be upstream of Physiological Load. Using data from the Indonesian Family Life Survey (IFLS), which had five survey waves from 1993 to 2015, we performed structural equation modeling (SEM) on two main models (Figure 2 and Figure 3). As the hypothesis concerns temporal effects, longitudinal data would be important. However, as only the latest survey wave (IFLS5) had all the indicators of interest, we first tested a comprehensive model (Model A) with cross-sectional data from IFLS5. This model includes all four Lifestyle Risk factors of interest. We then specified a second model (Model B) to allow the testing of longitudinal data (IFLS4 and IFLS5), but with only two of the Lifestyle Risk factors, as sleep and food intake were not collected in the earlier wave. We account for sociodemographic risk factors as covariates in these analyses.

## 2. Methods

### 2.1. Study Design and Respondents

In this retrospective cohort study, we used publically available data from the Indonesian Family Life Survey (IFLS), which has been organised by the RAND Corporation in collaboration with Lembaga Demografi, the University of Indonesia, Center for Population and Policy Studies, the University of Gadjah Mada, the University of California, Los Angeles, and SurveyMETER. A stratified random sampling scheme on the provinces was adopted and the resulting sample included 13 of 27 provinces in Indonesia, representative of 83% of the population. Households that were randomly selected and participated in the first survey in 1993 were followed up in subsequent waves. Sampling and survey methods have been discussed in detail elsewhere [26].

**Figure 4:**
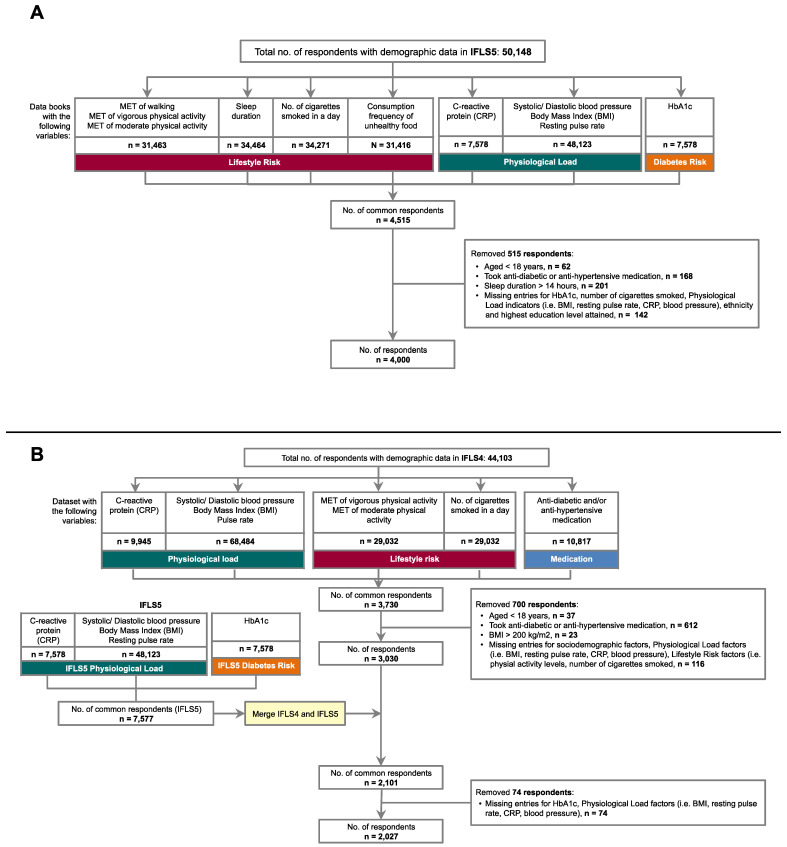
Flow charts of the study sampling for Model A (**A**) and Model B (**B**). Note: The number of respondents removed based on specific exclusion criteria do not add up to the total number removed, due to respondents who met multiple exclusion criteria.

We analysed data from Wave 4 (“IFLS4”, conducted from 2007–2008) and Wave 5 (“IFLS5”, conducted from 2014–2015), given that the indicators of interest were found in these two waves. Both waves had individual-level data on anthropometric measurements, morbidity indicators, healthcare utilization, health behaviours, employment, and household expenditure [26], though specific data on food frequency, sleep duration, and glycosylated haemoglobin (HbA1c) were not available in IFLS4. Figure 4 illustrates the data sampling for the two main models. To reduce the possible confounding effect of medication in this cross-sectional study, respondents who self-reported to be on anti-diabetic or anti-hypertensive medication were excluded. The final study sample for Model A included 4000 respondents aged 18 and above (12.9% of total 31,102 adult respondents in IFLS5). For Model B, it included 2027 respondents aged 18 and above (11.7% of the total 17,396 adult respondents present in IFLS4 and 5).

### 2.2. Sociodemographic Factors

Sociodemographic variables are important determinants in chronic disease and should be accounted for [27,28]. Age, sex, ethnicity, and highest educational level were specified as covariates separately for each of the main factors (Lifestyle Risk, Physiological Load, and Diabetes Risk) to account for confounding effects at any level.

Ethnicity was grouped into 3 categories: Javanese; Sundanese; and Others. Besides the Javanese and Sundanese, the other ethnicities each made up less than 6% of the sample population and thus were combined, similar to other studies [29,30].

The highest level of education attained was grouped into four categories following previous studies [9,29,31]: no education (unschooled); elementary (grade school, kindergarten, Islamic elementary school, adult education A); high school (general junior high, vocational junior high, Islamic Junior high, adult education B, general senior high, vocational senior high, Islamic senior high school, adult education C, Pesantren boarding school); and college/university (open university, Diploma, University).

### 2.3. Estimation of Lifestyle Risk

Four unhealthy lifestyle behaviours (physical inactivity, smoking, consumption frequency of unhealthy food, and insufficient sleep) were used as indicators of Lifestyle Risk (though in Model B, only physical inactivity and smoking were available for testing).

The level of physical activity in the last 7 days was assessed through the International Physical Activity Questionnaire (IPAQ). If respondents reported engaging in walking or any moderate or vigorous physical activities, the total duration of activities was converted to Metabolic Equivalent of Task (MET)-hours per week using the IPAQ recommended formula [32]. The amount of physical activity was reverse-coded and used as a measure of “physical inactivity”, which would contribute to Lifestyle Risk. The negatively phrased physical inactivity was used to maintain a consistent interpretation of Lifestyle Risk indicators.

The number of cigarettes smoked was used as an indicator for smoking intensity. This indicator was collected by questionnaires in IFLS4 and 5, which included questions on smoking status, whether the respondent has ever smoked self-rolled cigarettes, manufactured cigarettes, or cigars, followed by questions on smoking intensity, how many cigarettes/cigars smoked currently or before quitting. Respondents who reported “No” for questions on smoking status would have a smoking intensity of zero.

Unhealthy foods are highly processed food items that are higher in saturated fat, sugar, and sodium, such as instant noodles, sweet snacks, fried snacks, fast food, or soft drinks [33]. High consumption of such foods has been shown to exacerbate the risk of chronic diseases [34]. Therefore, for the purpose of this study, the consumption frequency of unhealthy foods in the past 7 days was used as an indicator of Lifestyle Risk. Unhealthy foods refer to instant noodles, sweet snacks, fried snacks, fast food, or soft drinks. (Food Frequency Section of IFLS5). The total score was calculated by summing the consumption frequencies of individual unhealthy food items in the past week with saturation at a score of seven, as the focus was on the consumption frequency of any type of unhealthy food within a week, therefore, the range of scores was 0–7.

Sleep duration was determined by two self-reported questions in IFLS5, the time the respondent slept the night before and the time the respondent woke up the day before the survey. The time respondent slept was subtracted by the time respondent woke up to compute sleep duration. Sleep duration was then reverse-coded and used as a measure of insufficient sleep, i.e., shorter sleep duration corresponded to more insufficient sleep. The negatively phrased insufficient sleep was used to maintain a consistent interpretation of Lifestyle Risk indicators.

To specify an appropriate structural equation model, we first assessed the suitability of Lifestyle Risk to be a latent construct within the model. A statistical check for an underlying latent construct requires its effect indicators to be positively correlated with one another [35]. However, we found weak correlation results for the indicators of Lifestyle Risk (Spearman’s rank *r* < |0.4|), showing that it would not be appropriate to create a Lifestyle Risk latent variable. See Table S1. The Lifestyle Risk indicators would be better identified as a composite variable because it does not violate this conceptual check [35]. Furthermore, the composite or cumulative effect of these high-risk lifestyle behaviours is associated with higher health risks [36]. As a composite score of high-risk lifestyle behaviours, this means that Lifestyle Risk can be estimated, rather than it being an underlying, unobservable construct.

As there are no standardized thresholds for risk levels in lifestyle behaviours, we used principal component analysis (PCA) to derive the weighted composites of the Lifestyle Risk indicators. The Lifestyle Risk indicators were included as continuous variables in the PCA models, and the principal components (i.e., weighted composites) were orthogonally transformed using Varimax rotation and underwent Kaiser normalization to achieve a structure with independent components for greater interpretability. Principal components with eigenvalue > 1, scree test, and parallel analysis were considered in determining the number of components to retain [37,38,39].

### 2.4. Estimation of Physiological Load

In this study, Physiological Load is conceptualised as an estimate of the cumulative physiological burden or stress on the body system, similar to the concept of allostatic load or “cumulative biological risk” [40]. Allostatic load describes the physiological consequences of the body’s attempts to adapt to chronic stressors, which may result in dysregulation spreading among multiple body systems, potentially combining to increase disease risk [40,41]. Summary measures are typically used to characterise allostatic load across the cardiovascular, metabolic, immune, nervous, and hormonal systems [40,42]. As this study does not focus on the nervous or hormonal systems, we have used the term, “Physiological Load”, based on five routine clinical monitoring indicators (body mass index, resting pulse rate, c-reactive protein, systolic and diastolic blood pressure) from IFLS4 and 5. Each of these Physiological Load indicators has been shown to be associated with diabetes [12,43,44,45,46,47,48].

The body mass index (BMI) was computed by taking the weight (kilograms) divided by the height (metres) squared. Resting pulse rate (RPR) and blood pressure were averaged from three measurements on alternate arms while respondents were seated upright. CRP concentrations were derived from finger prick dried blood samples and measured by a high-sensitivity CRP enzyme-linked immunosorbent assay (ELISA) [49]. CRP plasma equivalent values were used.

Prior to creating a summary measure of Physiological Load, we performed a conceptual check, similar to the one done for Lifestyle Risk, in order to ascertain suitability for specification as a latent construct. Except for SBP and DBP, which are closely related, low correlations, Spearman rank *r* < |0.4|, were found between the rest of the Physiological Load indicators, indicating that a latent variable would not be suitable [35] (See Table S1). We then proceeded to create a summary index of Physiological Load by using the method similar to allostatic load summary measurements: for each biomarker, a score of one is given for values beyond a clinical threshold reflecting high risk, with a score of zero given if otherwise [50]. These were then added up to form a non-weighted summary index (range of 0 to 5) of the Physiological Load for each respondent. High risk was defined as: BMI of ≥ 25 kg/m^2^ [51], RPR of ≥ 90 bpm [52], SBP ≥ 140 mmHg and DBP ≥ 90 mmHg [53]. CRP ≥ 30 mg/L (3 mg/dL) [54].

### 2.5. Estimation of Diabetes Risk

As the primary outcome variable, the risk of diabetes was estimated with the level of glycosylated haemoglobin (HbA1c), a surrogate biomarker of average glycemic control over the previous three months. HbA1c was assayed using dried blood samples from the IFLS respondents using a validated protocol [49,55] and was available only in IFLS5.

### 2.6. Structural Equation Modelling

Categorical variables (e.g., sex, ethnicity) were summarised through counts and percentages, while all the other variables were continuous and were summarised through minimum, maximum, median, and interquartile ranges (IQR).

Mediation analysis via SEM [56] was used to test the hypothesis that Lifestyle Risk has a positive but indirect effect on Diabetes Risk through Physiological Load mediators. In the models, weighted composites representing Lifestyle Risk were considered independent (exogenous) variables, Physiological Load was a mediator and HbA1c was the marker of the outcome variable of Diabetes Risk. Sociodemographic variables were controlled for as covariates of Lifestyle Risk, Physiological Load, and HbA1c [35].

Two main models were tested. Model A was a comprehensive model, encompassing all indicators of interest (Figure 2). We first tested the comprehensive model with cross-sectional data from IFLS5, as only IFLS5 had all indicators of interest. We then created a smaller model (Model B, Figure 3) that allowed us to do a longitudinal analysis with IFLS4 data. This model was similar to Model A, except for the Lifestyle Risk indicators of the frequency of unhealthy food consumption and sleep duration, both of which were unavailable in IFLS4. Physiological Load was modelled as the mean of Physiological Load from IFLS4 and 5, in order to obtain a value that would be temporally midway between the surveys of IFLS4 and 5. The continuous outcome variable of Diabetes Risk was represented by HbA1c values.

We used the maximum likelihood procedure that provides correction to estimates and standard errors, and a mean- and variance-adjusted chi-square test statistic robust to non-normality [57]. Standardized estimates with 95% confidence intervals (CI) and *p*-values were reported. Model fit was assessed with the root mean squared error of approximation (RMSEA), comparative fit index (CFI), Tucker-Lewis index (TLI), and standardized root mean squared residual (SRMR). The following values indicate acceptable model fits: RMSEA < 0.08, CFI and TLI > 0.8, and SRMR < 0.08 [58].

Data preparation and descriptive analyses were performed using STATA (version 14.0) software [59]. PCA and parallel analysis were performed on Rstudio (R version 4.0.2) software, using the *psych* (version 1.9.12) and *paran* (version 1.5.2) packages [60,61,62]. All mediation analyses were performed using Mplus 8 (version 1.5) software [63]. In all statistical analyses, *p* < 0.05 and 95% CI that do not include zero were considered statistically significant.

### 2.7. Post-Hoc Analyses

To investigate the possibility that the involvement of physical labour in respondents’ primary jobs confounded physical inactivity levels of Lifestyle Risk composite variables, physical labour was assessed by responses to two self-reported questions, whether respondents’ primary jobs involved physical effort or heavy lifting all the time, almost all the time, or most of the time. In this model (Model A2), involvement of physical labour in primary jobs was classified as “yes” or “no” based on their responses and adjusted for as a covariate of Lifestyle Risk.

To mitigate the weakness that the analysis for Model A used cross-sectional data, we compared the model fit results against an alternative model with reversed directional relationships (Model A3). The Bayesian information criterion (BIC) values were assessed, with the smaller BIC value indicating a better model fit.

## 3. Results

### 3.1. Sample Characteristics and Comparisons with the National Population

Table 1 presents a descriptive summary of demographic characteristics of the samples used in the analysis of Model A and Model B. It also includes summary statistics of the Lifestyle Risk indicators, Physiological Load indicators, and HbA1c levels.

The median age in the IFLS5 sample was 40 years (range 18–102 years) with 53.0% males. 59.5% of the respondents attained highest education level of high school and above (High school = 46.1%; College/university = 13.4%). This is similar to the Indonesian population, where 50.3% of the national population were males, the two largest ethnic groups were also Javanese and Sundanese, and 54.9% of the population attained an education level of at least high school and above [30,64,65,66].

The IFLS4 sample had some different characteristics. While the Javanese and Sundanese remained the two largest ethnic groups, the median age was 55 years (range 23–88 years) with 43.4% males, and the highest level of education for the majority was elementary school (56.2%). Differences in these factors between the IFLS4 sample and the national statistics can be attributed to the filtering of respondents that did not meet the inclusion criteria as outlined in the Methods (see Figure 4). Differences between the IFLS4 and IFLS5 samples could also be attributed to change in demographic progression across the 7–8 years between Waves 4 and 5, such as the increase in the proportion of those with higher education.

**Figure 5:**
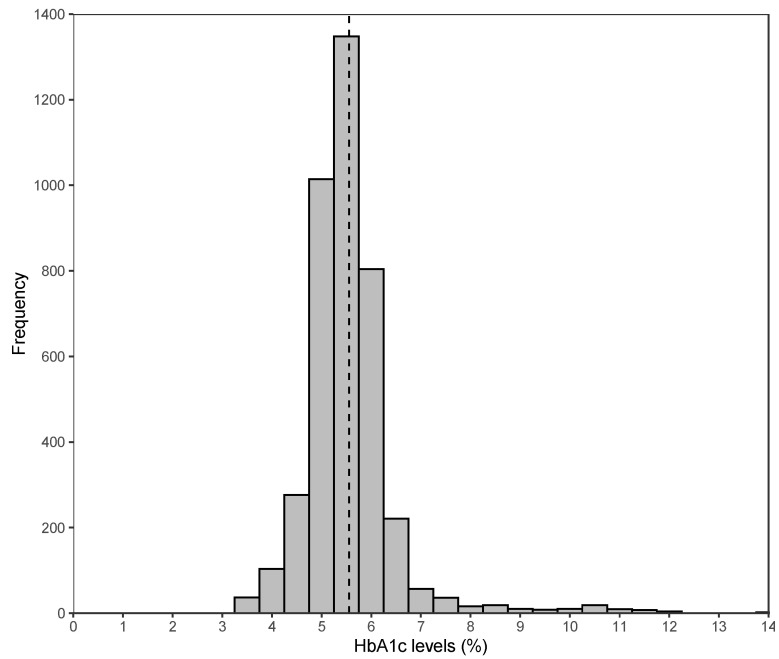
Distribution of HbA1c levels (%) among the IFLS5 sample (4000 respondents aged 18 and above). Note: Dashed line indicates the mean at 5.55%. The minimum was 3.50% and the maximum was 14.0%.

Figure 5 illustrates the distribution of the HbA1c level among the respondents. The median HbA1c level was 5.45% (range 3.50–14.0%). Applying the World Health Organisation (WHO) cut-off for indication of diabetes, i.e., HbA1c ≥ 6.50% [67], 6.83% of our respondents were diabetic (see Table 1), which is very similar to the 6.90% national diabetes prevalence reported in 2013 Indonesia Basic Health Research survey (RISKESDAS) [68], indicating representativeness of our sample.

### 3.2. Model A

Model A was tested with IFLS5 data. Following PCA to determine the composite weights of Lifestyle Risk, we selected the first two components for Lifestyle Risk data upon inspection of the scree plot, applying the Kaiser-Guttman rule, and parallel analysis. The two components were termed LR1 and LR2. The variance explained by LR1 and LR2 was 28.27% and 26.63%, respectively. The total proportion of variance explained by the selected components was 54.9% for IFLS5 data.

Table 2 presents the retained components and their loadings. The strong loadings in LR1 were physical inactivity (−0.75) and smoking (0.74), while the strong loadings in LR2 were consumption frequency of unhealthy food (0.72) and insufficient sleep (0.71). All component loadings were positive, except for physical inactivity (−0.75) and the consumption frequency of unhealthy food (−0.10) in LR1.

Table 3 presents the direct and indirect relationships between Lifestyle Risk and Diabetes Risk (HbA1c). The model had good fits (RMSEA < 0.05; CFI and TLI > 0.95; SRMR < 0.08). Both components of Lifestyle Risk (LR1 and LR2) were shown to have effects on Diabetes Risk through the mediator of Physiological Load. LR1 did not have a direct effect, but there was an indirect effect on Diabetes Risk, through an inverse relationship with Physiological Load. LR2 had both positive direct and indirect effects on Diabetes Risk through Physiological Load. For the detailed results, including statistics for the sociodemographic covariates, refer to Table S2.

### 3.3. Model B

Model B was tested with longitudinal data from IFLS4-5. Following PCA to determine Lifestyle Risk, we also selected the first two components (LR1 and LR2), as they passed the Kaiser-Guttman rule, though parallel analysis recommended retaining only the first component. The positive loadings of the second component were also in line with our hypothesis. LR1 and LR2 explained 55.97% and 44.03% of variance, respectively. The loadings of LR1 were similar to that of Model A (Table 2), where the loadings for physical inactivity and smoking were −0.75 and 0.75, respectively. The loadings of LR2 were 0.66 for both physical inactivity and smoking.

The model had acceptable fits (RMSEA < 0.08; CFI > 0.95; SRMR < 0.08) (See Table 3). The first Lifestyle Risk component, LR1, did not have a significant effect on Physiological Load or Diabetes Risk. However, the second Lifestyle Risk component, LR2, had a positive indirect effect on Diabetes Risk through positive effects on Physiological Load. For detailed results, including statistics for the sociodemographic covariates, refer to Table S3.

### 3.4. Comparisons with Alternative Models

With reference to physical activity guidelines [69,70], at least 8.30 MET hours/week of physical activity is recommended. Compared to this, the medians for the IFLS4 and IFLS5 samples were higher by approximately 6 times (49.0 MET hours/week) and 4 times (31.5 MET hours/week) respectively (Table 1). Due to the surprisingly robust physical activity levels, we postulated that these levels could be confounded by jobs that involved physical labour. Indeed, individuals with physical labour in their primary jobs had significantly higher MET levels than individuals who did not, *t*(3998) = 13.1, *p* < 0.001, indicating that the jobs with manual labour contributed to this “lifestyle behaviour”. Therefore, the involvement of physical labour in respondents’ primary jobs was adjusted for as a covariate of Lifestyle Risk composite variables in a post-hoc analysis (Model A2). There was a positive and significant association between the involvement of physical labour and the Lifestyle Risk composite variable of LR1 (0.123, *p* < 0.001), but not for LR2. Otherwise, the standardized estimates and significance of direct and indirect relationships in the model were similar to Model A, with model fit indices indicating good fit (RMSEA < 0.05; CFI > 0.95; TLI > 0.80; SRMR < 0.08) (see Table S4).

Given that Model A was based on cross-sectional data, we compared it to a model in which the pathways were reversed (Model A3). BIC values were lower for Model A (42,703.629) than Model A3 (42,709.770), indicating a better fit for Model A.

### 3.5. Sociodemographic Covariates

Results of the sociodemographic covariates can be found in Figure 2 and Figure 3, and Tables S2 and S3. The significant covariates were mostly consistent between the models. In summary, increasing age was associated with increasing Physiological Load. Males were associated with poorer lifestyles, especially in terms of diet and sleep (LR2). Higher education was associated with higher Lifestyle Risk and Physiological Load.

## 4. Discussion

We have developed a general pathway model from the start point of modifiable lifestyle behaviour and have demonstrated how the behavioural components in Lifestyle Risk can affect Diabetes Risk via the mediating factor of Physiological Load. The overall results support our hypothesis that the effect of Lifestyle Risk on Diabetes Risk is likely to be indirect, and thus offers a stepwise perspective, whereby upstream and downstream modifiable factors could be modeled pathwise. Specifying Lifestyle Risk as an observable composite variable incorporates the cumulative effect of risk behaviour and differentiates this study from previous studies looking at it as a latent construct [16,71,72]. There was also the advantage of being able to assess causality using seven-year follow-up data, albeit only for Model B. From a disease prevention perspective, it helps to narrow our focus to an initial set of lifestyle risk factors, from which to monitor the progression of health risk towards more downstream physiological factors to disease.

The use of mediation analysis via SEM allowed us to simultaneously assess multiple pathways within a single model, in addition to accommodating a variable to be both independent and dependent (i.e., a mediator). This confers advantages over traditional regression, where multiple pathways in a single model need to be tested separately, resulting in potential problems with multiple comparisons [73]. To date, only a few studies have simultaneously analysed risk factors in diabetes in a multiple pathway system. Bardenheier et al., 2013 [16] performed the first study, using 10 variables with 27 hypothesised pathways in an SEM and found through best-fit iterations of the model that physical activity and poor diet were significant lifestyle factors (other lifestyle factors were not studied) that contributed to diabetes risk via large waist circumference, high blood pressure, triglycerides, and high-density lipoprotein (HDL). Subsequently several other studies applied similar models in their own population data [71,72] with varying results, but generally finding that physical activity and poor diet impact diabetes risk through separate mediators like BMI, blood pressure, HDL, and triglycerides. One difference between the earlier studies and this study is the use of latent constructs to model lifestyle behaviour in the earlier studies. As latent variables are meant to be unobservable constructs [35], a behaviour such as physical activity may not be suitable to be modeled as a latent construct, since it is actually observable. It would be necessary to evaluate the assumption for latent variables by testing if the effect indicators are correlated, a result that we did not find in our analysis (Table S1). We thus modelled Lifestyle Risk as a composite variable comprising linear, weighted combinations of risk from the uncorrelated lifestyle behaviours [35].

### 4.1. Components of Lifestyle Risk

In both models, two Lifestyle Risk components (LR1 and LR2) were derived from the data as complementary composites of unhealthy behaviour. PCA as a data-driven approach was used because there are currently no standardized thresholds for risk levels in lifestyle behaviours. The LR2 component encompassed a straightforward set of unhealthy lifestyle behaviours (all behaviour indicators had positive coefficients). As hypothesised, an increase in Lifestyle Risk goes on to increase Diabetes Risk through the mediator of Physiological Load. Physical activity and poor diet are both well established risk factors corresponding to the risk of chronic disease, in particular diabetes [74,75,76]. Smoking is also another risk factor, showing a dose-response phenomenon with risk of diabetes [77], while short sleep duration is gaining attention as a factor involved in developing risk of diabetes through associations with BMI and blood pressure [78,79,80]. The path coefficients from LR2 to Physiological Load were relatively small (0.04–0.05), though the effect sizes were within the range of a study that used regressions to assess lifestyle variables and BMI with the same IFLS dataset [81]. While the indirect effect of LR2 on Diabetes Risk was clear in both models, the direct effect was uncertain, given that this effect was found in Model A but not in Model B. However the use of longitudinal data to test Model B lends weight to its results and aligns with studies showing the indirect effects of physical activity on the risk of developing diabetes through intermediate variables like BMI, but without detected direct effects [16,73,82].

The other lifestyle risk component, LR1, is an intriguing mix of behaviour found in both models: the composite weightings for physical inactivity and smoking are in opposite directions, i.e., being physically active while smoking contributes to LR1. This apparent contradiction in lifestyle may be explained by our post hoc analysis showing that a significant contributor to the high physical activity scores in the sample is involvement in physical labour as part of work, rather than recreational exercise. The effects of LR1 on Diabetes Risk were equivocal, as there was a significant indirect effect in Model A, but not in Model B. We lean towards the results of Model B, given the longitudinal testing. The factor of time may explain the different results, for example, it has been shown that the number of cigarettes smoked appears to have a negative correlation with the risk of high blood pressure, but when adjusted for life-course, the correlation turned positive [36]. In the longitudinal Model B, the negative effects of smoking may have over time counterbalanced the protective effects of physical activity, thus nullifying any overall effect of LR1 on Diabetes Risk.

### 4.2. Physiological Load as a Mediator of Diabetes Risk

Physiological Load was found to be a significant mediator in both main models, with every unit increase in Physiological Load corresponding to an increase in HbA1c value by approximately 0.2 percentage points (based on the unstandardised estimates). (A unit increase in Physiological Load can be achieved by a cross in the clinical threshold for any of the five indicators, which are also used in routine clinical monitoring.) The pathway coefficients are within the ranges found in studies relating individual physiological markers to diabetes risk [16,71,72]. Furthermore, each of them have been associated with the effects of lifestyle behaviour [7,19,21,83,84,85,86], and thus may be considered intermediate markers of diabetes risk. The measure of Physiological Load is a subset of the allostatic load summary measure [40], as we focused on using typical clinical routine monitoring indicators in the metabolic and cardiovascular domains and did not include indicators of nervous and hormonal responses to chronic stress [40,41,42]. There is growing research that points to the utility of using grouped measures of physiological indicators to predict clinical risk, for example, the presence of adverse risk factors across multiple physiological systems strongly predicts morbidity and mortality [87]. A higher allostatic load has been found in patients with Type 2 diabetes [88,89] and correlates with higher glycated haemoglobin [89]. Physiological dysfunction can also spread across multiple physiological systems and combine to elevate disease risk [42,50]. Importantly, the grouped measures of physiological dysregulation appear to better predict morbidity and mortality risks as compared to individual risk indicators [50,90,91].

### 4.3. Diabetes Risk

In this large sample, 6.83% of respondents had HbA1c values of 6.50% and above and can be classified diabetic, according to WHO criteria [67]. This statistic is very similar to the 6.90% found in a national health survey in Indonesia done around the same time [68], indicating the representativeness of our sample. We observed that 90% of survey respondents with HbA1c values of ≥6.50% did not report themselves as having received a diagnosis of diabetes (see Table S5). The incongruence of high HbA1c values with a low incidence of awareness/reported diagnosis is a concern for public health efforts, notwithstanding the disease burden from known cases, being one of the top five countries with diabetes cases [2].

The availability of continuous HbA1c values in the IFLS data was well-suited for the performing of SEM and conferred an advantage over studies using categorical or binary diabetes outcomes, as unbalanced samples may require the employment of correction techniques, such as oversampling [24]. Besides yielding greater statistical power and precision, having a single continuous outcome reduces the number of parameters in the model, contributing to its parsimony [92].

### 4.4. Applications and Limitations

As the purpose of the general model is to provide a simple pathway framework that groups distal (lifestyle) and proximal (physiological) factors, it can be applied to any analysis concerned with evaluating the relationship of lifestyle risks with chronic disease via the mediator of physiological risk. Specific composites of the distal or proximal factors can be determined by knowledge of the disease etiology, or driven by the dataset at hand, such as was done with the Lifestyle Risk indicators in this study.

Food frequency and sleep duration were lifestyle indicators important to our model, but as they were only found in IFLS Wave 5, the comprehensive model (Model A) was constrained to using the available cross-sectional data, which limited causal attribution. We assumed, similar to other studies (e.g., Bardenheier et al., 2013 [16]), that the reported lifestyle behaviour was habitual, built up over time, and thus preceded Physiological Load and Diabetes Risk. We mitigated the issue of cross-sectional data analysis in three ways. First, we excluded all the respondents who reported that they were taking medication for diabetes and hypertension, in order to avoid the confounding effect of medication on the Physiological Load indicators. Second, we generated a model with all pathways reversed to compare with our hypothesised model, in order to check that the hypothesised model was the better fit, which was shown to be the case. Third, we did a longitudinal analysis using a smaller model (Model B), without the Lifestyle Risk indicators of food and sleep. This longitudinal analysis supported the mediation effect found in the comprehensive model.

A potential confounding factor for the longitudinal analysis is the changing exposure to health policies and health promotion programs during the seven to eight years in between survey waves. The decentralized, district-level approach in Indonesian healthcare [93] precludes straightforward adjustments of effects to program exposure, since the IFLS respondents would not have had uniform exposure to programs, being from districts across Indonesia. The interpretation of the cross-sectional analysis for Model A remains unaffected.

The self-reports for lifestyle behaviour in the study pose a potential weakness, as the Lifestyle Risk indicators are subject to measurement errors and self-recall biases. As wearables and fitness trackers become more commonplace in the future, health behaviour data from such devices would form more objective sources for feeding into the model.

## 5. Conclusions

We have presented a general model illustrating modifiable pathways from Lifestyle Risk to Diabetes Risk via the mediating factor of Physiological Load and have tested it using large datasets from Wave 4 and Wave 5 of the Indonesian Family Life Survey. Non-modifiable sociodemographic covariates were accounted for in the model, while focusing on what is amenable for health outcomes. The model illustrates parsimonious and modifiable pathways that could be applied in public health efforts for diabetes or chronic disease prevention.

## Figures and Tables

**Table 1:** Sociodemographic, lifestyle and physiological characteristics.

	IFLS5 Sample			IFLS4 Sample		
Characteristics	n (%) or Median (IQR)		Proportion of Sample beyond Clinical Threshold	n (%) or Median (IQR)		Proportion of Sample beyond Clinical Threshold
**Age**, median (IQR)	40.00	(31.0, 56.0)	-	55.00	(49.0, 62.0)	-
**Sex**, n (%)						
Male	2078	(53.0%)	-	879	(43.4%)	-
Female	1854	(47.0%)	-	1148	(56.6%)	-
**Ethnicity**, n (%)						
Javanese	1853	(47.6%)	-	982	(48.4%)	-
Sundanese	425	(10.8%)	-	228	(11.2%)	-
Others	1654	(41.6%)	-	817	(40.3%)	-
**Highest Education level**, n (%)						
No education	235	(5.93%)	-	360	(17.8%)	-
Elementary	1360	(34.6%)	-	1139	(56.2%)	-
High school	1810	(46.1%)	-	424	(20.9%)	-
College/University	527	(13.4%)	-	104	(5.13%)	-
**Lifestyle Risk indicators**, median (IQR)						
Physical activity levels (MET h/w)	31.54	(8.25, 86.7)	-	48.96	(17.0, 113.0)	-
Smoking (cigarettes smoked in a day)	0.00	(0.00, 10.0)	-	0.00	(0.00, 6.00)	-
Consumption frequency of unhealthy food (days in a week)	5.00	(2.00, 7.00)	-	-	-	-
Sleep duration (h)	7.00	(5.67, 8.00)	-	-	-	-
**Physiological Load indicators**, median (IQR)						
BMI (kg/m^2^)	22.56	(19.9, 25.9)	30.7%	22.12	(19.7, 25.4)	26.9%
Resting pulse rate (bpm)	74.67	(68.0, 82.3)	9.58%	75.33	(69.3, 82.0)	10.2%
CRP (mg/L)	0.75	(0.263, 2.05)	0.375%	0.84	(0.350, 1.99)	0.00%
Systolic blood pressure (mmHg)	125.67	(116, 138)	22.9%	136.00	(124, 152)	34.9%
Diastolic blood pressure (mmHg)	78.00	(71.7, 85.7)	14.9%	80.17	(73.8, 87.5)	18.8%
**Diabetes Risk**, median (IQR)						
HbA1c level (% level)	5.45	(5.10, 5.85)	6.83%	-	-	-

Abbreviations: Indonesian Family Life Survey Wave 4/Wave 5 (IFLS4/IFLS5); Interquartile Range (IQR); Metabolic Equivalent of Task (MET); Body Mass Index (BMI); C-reactive protein (CRP); Glycosylated haemoglobin (HbA1c); Descriptive statistics are presented in n (%) for categorical variables and median (interquartile range) for continuous variables. All values with decimals were rounded off to 3 significant figures. Age statistics were calculated based on respondents’ ages in year 2015 for IFLS5 and respondents’ ages in year 2008 for IFLS4. Physical activity and sleep duration were later reverse-coded by multiplying with (−1) to maintain a consistent negative interpretation of all Lifestyle Risk indicators. Clinical thresholds for Physiological Load indicators were BMI ≥ 25 kg/m^2^; resting pulse rate ≥ 90 bpm; c-reactive protein (CRP) ≥ 30 mg/L; systolic blood pressure ≥ 140 mmHg; diastolic blood pressure ≥ 90 mmHg; HbA1c ≥ 6.50%.

**Table 2:** Component matrix of the 2-component principal component analysis (PCA).

Lifestyle Risk Indicators (Model A)	LR1	LR2
Physical inactivity	−0.75	0.12
Smoking	0.74	0.14
Consumption frequency of unhealthy food	−0.10	0.72
Insufficient sleep	0.13	0.71
**Lifestyle Risk Indicators (Model B)**	**LR1**	**LR2**
Physical inactivity	−0.75	0.66
Smoking	0.75	0.66

Abbreviations: Lifestyle Risk component 1 (LR1); Lifestyle Risk component 2 (LR2); Principal components were orthogonality transformed using varimax rotation. All values were rounded off to 2 decimal places.

**Table 3:** Model A and Model B with standardized estimates, *p*-values and 95% confidence interval.

		Model A				Model B			
		Standardized Estimate	*p*-Value	95% CI		Standardized Estimate	*p*-Value	95% CI	
Direct effects on Diabetes Risk									
	LR1	−0.012	0.461	(−0.045,	0.020)	0.007	0.817	(−0.054,	0.069)
	LR2	**0.033**	<0.050	(0.002,	0.064)	0.032	0.244	(−0.022,	0.086)
Indirect effects on Diabetes Risk via Physiological Load mediator									
	LR1	**−0.011**	<0.050	(−0.019,	−0.004)	−0.003	0.633	(−0.013,	0.008)
	LR2	**0.009**	<0.050	(0.003,	0.016)	**0.010**	<0.050	(0.001,	0.020)
									
Model fit indices	RMSEA	0.014				0.069			
	CFI	1.000				0.988			
	TLI	0.989				0.601			
	SRMR	0.002				0.016			

Abbreviations: Lifestyle Risk component 1 (LR1); Lifestyle Risk component 2 (LR2); Root mean squared error of approximation (RMSEA); Comparative fit index (CFI); Tucker-Lewis index (TLI); Standardized root mean squared residual (SRMR); Significant estimates at *p* < 0.05 are shown in bold. All values were rounded off to 3 decimal places. Model A (using IFLS5 data) adjusted for sociodemographic covariates: age, sex, ethnicity, and highest education level attained in IFLS5 (full model results in Table S2). Model B (using IFLS4 and 5 data) adjusted for sociodemographic covariates: age, sex, ethnicity, and highest education level attained in IFLS4 (full model results in Table S3). Statistically significant estimates (in bold) showed Physiological Load mediated the indirect effects of Lifestyle Risk (LR2) on Diabetes Risk. Model fit indices were within acceptable thresholds.

## Data Availability

Publicly available datasets were analyzed in this study. This data can be found here: https://www.rand.org/well-being/social-and-behavioral-policy/data/FLS/IFLS/access.html (accessed on 25 August 2021).

## Supplementary Materials

The following are available online at www.mdpi.com/article/10.3390/ijerph182010907/s1. Table S1: Spearman correlation matrix of Lifestyle Risk and Physiological Load indicators; Table S2: Model A—Full statistics of all direct and indirect relationships; Table S3: Model B—Full statistics of all direct and indirect relationships; Table S4: Model A2—To assess the inclusion of physical labour as a covariate; Table S5: Comparison of Diabetes prevalence determined by self-reports and HbA1c level.

## List of Abbreviations

95% CI	95% confidence intervals
BIC	Bayesian Information Criterion
BMI	Body Mass Index
CFI	Comparative Fit Index
CRP	C-reactive protein
DALY	Disability-Adjusted Life Years
DBP	Diastolic blood pressure
HbA1c	Glycosylated haemoglobin
HDL	High-density lipoprotein
IFLS(4/5)	Indonesian Family Life Survey (Wave 4/Wave 5)
IPAQ	International Physical Activity Questionnaire
IQR	Interquartile range
LR1	Lifestyle Risk component 1
LR2	Lifestyle Risk component 2
MET	Metabolic Equivalent of Task
PCA	Principal component analysis
RMSEA	Root mean squared error of approximation
RPR	Resting pulse rate
SBP	Systolic blood pressure
SEM	Structural equation modelling
SRMR	Standardized root mean squared residual
TLI	Tucker-Lewis Index
WHO	World Health Organisation
