# Supplementary material for: Towards a Parsimonious Pathway Model of Modifiable and Mediating Risk Factors Leading to Diabetes Risk
Source: Int J Environ Res Public Health. 2021 Oct 17;18(20):10907. doi: 10.3390/ijerph182010907 (PMC8536137; doi:10.3390/ijerph182010907)
Supplement: Supplementary file 1 [file ijerph-18-10907-s001.zip › SupplementaryFiles/TableS5.pdf]

Table S5. Comparison of Diabetes prevalence determined by self-reports and HbA1c level

		Self-reported Diabetes diagnosis		
		**No**	**Yes**	Total
**HbA1c ≥ 6.50%**	**No**	3712	15	3727
	**Yes**	246	27	273
	Total	3958	42	4000

1. Self-reports of previous diagnosis by doctors or nurses (yes/no) were used for comparison.
2. HbA1c cut-off of 6.50% (yes/no) was used for comparison.
