# Supplementary material for: Towards a Parsimonious Pathway Model of Modifiable and Mediating Risk Factors Leading to Diabetes Risk
Source: Int J Environ Res Public Health. 2021 Oct 17;18(20):10907. doi: 10.3390/ijerph182010907 (PMC8536137; doi:10.3390/ijerph182010907)
Supplement: Supplementary file 1 [file ijerph-18-10907-s001.zip › SupplementaryFiles/TableS4.pdf]

Table S4. Model A2 - To assess the inclusion of physical labour as a covariate.

		Standardized estimate	*p*-value	95% CI
**Direct effects**				
LR1				
	Involvement in physical labour	**0.123**	< 0.001	(0.098, 0.148)
	Age (in year 2015)	**0.040**	< 0.050	(0.008, 0.072)
	Sex			
	Male	*Ref*		
	Female	**-0.509**	< 0.001	(-0.529, -0.488)
	Ethnicity			
	Javanese	*Ref*		
	Sundanese	**-0.041**	< 0.050	(-0.066, -0.015)
	Others	-0.014	0.312	(-0.042, 0.013)
	Highest education level			
	No education	*Ref*		
	Elementary	-0.044	0.116	(-0.100, 0.011)
	High school	**-0.106**	< 0.050	(-0.168, -0.043)
	College/University	**-0.144**	< 0.001	(-0.190, -0.099)
LR2				
	Involvement in physical labour	0.003	0.834	(-0.027, 0.033)
	Age (in year 2015)	0.036	0.054	(-0.001, 0.073)
	Sex			
	Male	*Ref*		
	Female	**-0.073**	< 0.001	(-0.103, -0.043)
	Ethnicity			
	Javanese	*Ref*		
	Sundanese	**0.084**	< 0.001	(0.055, 0.113)
	Others	**-0.127**	< 0.001	(-0.159, -0.095)
	Highest education level			
	No education	*Ref*		
	Elementary	**0.172**	< 0.001	(0.107, 0.238)
	High school	**0.331**	< 0.001	(0.258, 0.404)
	College/University	**0.320**	< 0.001	(0.267, 0.373)
Physiological Load mediator				
	LR1	**-0.053**	< 0.050	(-0.088, -0.019)
	LR2	**0.045**	< 0.050	(0.015, 0.076)
	Age (in year 2015)	**0.251**	< 0.001	(0.218, 0.285)
	Sex			
	Male	*Ref*		
	Female	**0.106**	< 0.001	(0.071, 0.142)
	Ethnicity			
	Javanese	*Ref*		
	Sundanese	-0.004	0.785	(-0.036, 0.027)
	Others	-0.023	0.148	(-0.055, 0.008)
	Highest education level			
	No education	*Ref*		
	Elementary	0.060	0.088	(-0.009, 0.128)
	High school	0.060	0.121	(-0.016, 0.135)
	College/University	**0.059**	< 0.050	(0.002, 0.116)
HbA1c				
	LR1	-0.012	0.460	(-0.045, 0.020)
	LR2	**0.033**	< 0.050	(0.002, 0.064)
	Physiological Load mediator	**0.207**	< 0.001	(0.171, 0.244)
	Age (in year 2015)	**0.144**	< 0.001	(0.108, 0.180)
	Sex			

		Standardized estimate	*p*-value	95% CI
	Male	*Ref*		
	Female	**-0.071**	< 0.001	(-0.107, -0.035)
	Ethnicity			
	Javanese	*Ref*		
	Sundanese	-0.003	0.875	(-0.034, 0.029)
	Others	0.018	0.276	(-0.014, 0.049)
	Highest education level			
	No education	*Ref*		
	Elementary	-0.047	0.218	(-0.121, 0.028)
	High school	-0.022	0.603	(-0.107, 0.062)
	College/University	-0.027	0.398	(-0.089, 0.035)
Intercepts	LR1	**0.436**	< 0.001	(0.255, 0.617)
	LR2	**-0.514**	< 0.001	(-0.726, -0.302)
	Physiological Load mediator	0.207	0.355	(-0.291, 0.104)
	HbA1c	**5.447**	< 0.001	(5.039, 5.856)
Residual variances	LR1	**0.695**	< 0.001	(0.673, 0.716)
	LR2	**0.913**	< 0.001	(0.896, 0.930)
	Physiological Load mediator	**0.92**	< 0.001	(0.904, 0.936)
	HbA1c	**0.923**	< 0.001	(0.905, 0.940)
**Indirect effects on HbA1c via Physiological Load mediator**				
HbA1c				
	LR1	**-0.011**	< 0.050	(-0.019, -0.004)
	LR2	**0.009**	< 0.050	(0.003, 0.016)
R^2	LR1	0.305		
	LR2	0.087		
	Physiological Load mediator	0.08		
	HbA1c	0.077		
Model fit indices	RMSEA	0.031		
	CFI	0.995		
	TLI	0.941		
	SRMR	0.007		

Significant estimates at p < 0.05 are shown in bold. All values were rounded off to 3 decimal places.
1. Model fit indices indicated that the model has generally good fits.
