# Supplementary material for: Towards a Parsimonious Pathway Model of Modifiable and Mediating Risk Factors Leading to Diabetes Risk
Source: Int J Environ Res Public Health. 2021 Oct 17;18(20):10907. doi: 10.3390/ijerph182010907 (PMC8536137; doi:10.3390/ijerph182010907)
Supplement: Supplementary file 1 [file ijerph-18-10907-s001.zip › SupplementaryFiles/TableS1.pdf]

Table S1. Spearman correlation matrix of Lifestyle Risk and Physiological Load indicators.

Indicators	Total physical inactivity levels	Smoking	Consumption frequency of unhealthy food	Insufficient sleep	BMI	Resting pulse rate	CRP	Systolic blood pressure	Diastolic blood pressure
Total physical inactivity levels	1.000								
Smoking	-0.121	1.000							
Consumption frequency of unhealthy food	0.008	0.000	1.000						
Insufficient sleep	-0.029	0.067	0.067	1.000					
BMI	0.058	-0.218	0.065	0.079	1.000				
Resting pulse rate	0.105	-0.117	0.068	0.001	0.196	1.000			
CRP	0.058	-0.098	0.038	-0.006	0.364	0.212	1.000		
Systolic blood pressure	-0.042	0.076	-0.098	0.059	0.208	0.079	0.082	1.000	
Diastolic blood pressure	0.022	-0.029	-0.025	0.060	0.329	0.216	0.138	0.756	1.000

BMI: body mass index; CRP: c-reactive protein. All values were rounded off to 3 decimal places.

1. Physical activity and sleep duration were reverse-coded by multiplying with (-1) to physical inactivity levels and insufficiant sleep respectively, to maintain a consistent negative interpretation of all Lifestyle Risk indicators.

2. All Spearman's rank correlation, $r < |0.4|$, indicated that the indicators are poorly correlated.
