# Supplementary material for: Towards a Parsimonious Pathway Model of Modifiable and Mediating Risk Factors Leading to Diabetes Risk
Source: Int J Environ Res Public Health. 2021 Oct 17;18(20):10907. doi: 10.3390/ijerph182010907 (PMC8536137; doi:10.3390/ijerph182010907)
Supplement: Supplementary file 1 [file ijerph-18-10907-s001.zip › SupplementaryFiles/TableS3.pdf]

Table S3. Model B - Full statistics of all direct and indirect relationships.

		Standardized estimate	*p*-value	95% CI
**Direct effects**				
LR1 (IFLS4)				
	Age (in year 2015)	**-0.118**	< 0.001	(-0.155, -0.081)
	Sex			
	Male	*Ref*		
	Female	**-0.558**	< 0.001	(-0.596, -0.520)
	Ethnicity			
	Javanese	*Ref*		
	Sundanese	-0.024	0.168	(-0.058, 0.010)
	Others	-0.011	0.582	(-0.050, 0.028)
	Highest education level			
	No education	*Ref*		
	Elementary	-0.012	0.611	(-0.060, 0.035)
	High school	**-0.065**	< 0.050	(-0.114, -0.016)
	College/University	**-0.094**	< 0.001	(-0.135, -0.053)
LR2 (IFLS4)				
	Age (in year 2015)	**0.075**	< 0.050	(0.032, 0.117)
	Sex			
	Male	*Ref*		
	Female	**-0.269**	< 0.001	(-0.313, -0.225)
	Ethnicity			
	Javanese	*Ref*		
	Sundanese	**0.046**	< 0.050	(0.007, 0.085)
	Others	**0.133**	< 0.001	(0.091, 0.176)
	Highest education level			
	No education	*Ref*		
	Elementary	0.003	0.916	(-0.051, 0.056)
	High school	**0.102**	< 0.001	(0.046, 0.158)
	College/University	**0.085**	< 0.001	(0.040, 0.130)
Physiological Load mediator				
	LR1 (IFLS4)	-0.012	0.633	(-0.062, 0.038)
	LR2 (IFLS4)	**0.048**	< 0.050	(0.005, 0.092)
	Age (in year 2015)	**0.067**	< 0.050	(0.023, 0.112)
	Sex			
	Male	*Ref*		
	Female	**0.214**	< 0.001	(0.163, 0.265)
	Ethnicity			
	Javanese	*Ref*		
	Sundanese	0.022	0.340	(-0.023, 0.068)
	Others	**-0.066**	< 0.050	(-0.110, -0.022)
	Highest education level			
	No education	*Ref*		
	Elementary	**0.062**	< 0.050	(0.004, 0.120)
	High school	**0.107**	< 0.001	(0.047, 0.166)
	College/University	**0.139**	< 0.001	(0.086, 0.193)
HbA1c (IFLS5)				
	LR1 (IFLS4)	0.007	0.817	(-0.054, 0.069)
	LR2 (IFLS4)	0.032	0.244	(-0.022, 0.086)

		Standardized estimate	*p*-value	95% CI
	Physiological Load mediator	**0.215**	< 0.001	(0.168, 0.262)
	Age (in year 2015)	-0.027	0.235	(-0.071, 0.017)
	Sex			
	Male	*Ref*		
	Female	0.001	0.986	(-0.060, 0.061)
	Ethnicity			
	Javanese	*Ref*		
	Sundanese	**-0.040**	< 0.050	(-0.076, -0.003)
	Others	0.018	0.457	(-0.029, 0.064)
	Highest education level			
	No education	*Ref*		
	Elementary	0.006	0.840	(-0.052, 0.063)
	High school	0.051	0.093	(-0.008, 0.111)
	College/University	0.019	0.458	(-0.030, 0.068)
Intercepts	LR1 (IFLS4)	**1.426**	< 0.001	(1.148, 1.703)
	LR2 (IFLS4)	**-0.337**	< 0.050	(-0.666, -0.007)
	Physiological Load mediator	**0.215**	< 0.050	(0.003, 0.661)
	HbA1c (IFLS5)	**4.959**	< 0.001	(4.466, 5.451)
Residual variances	LR1 (IFLS4)	**0.696**	< 0.001	(0.657, 0.735)
	LR2 (IFLS4)	**0.883**	< 0.001	(0.854, 0.912)
	Physiological Load mediator	**0.942**	< 0.001	(0.923, 0.962)
	HbA1c (IFLS5)	**0.947**	< 0.001	(0.926, 0.968)
**Indirect effects on HbA1c (IFLS5) via Physiological Load mediator**				
HbA1c (IFLS5)				
	LR1 (IFLS4)	-0.003	0.633	(-0.013, 0.008)
	LR2 (IFLS4)	**0.010**	< 0.050	(0.001, 0.020)
R^2	LR1 (IFLS4)	0.304		
	LR2 (IFLS4)	0.117		
	Physiological Load mediator	0.058		
	HbA1c (IFLS5)	0.053		
Model fit indices	RMSEA	0.069		
	CFI	0.988		
	TLI	0.601		
	SRMR	0.016		

Significant estimates at $p < 0.05$ are shown in bold. All values were rounded off to 3 decimal places.
