# Supplementary material for: Towards a Parsimonious Pathway Model of Modifiable and Mediating Risk Factors Leading to Diabetes Risk
Source: Int J Environ Res Public Health. 2021 Oct 17;18(20):10907. doi: 10.3390/ijerph182010907 (PMC8536137; doi:10.3390/ijerph182010907)
Supplement: Supplementary file 1 [file ijerph-18-10907-s001.zip › SupplementaryFiles/TableS2.pdf]

Table S2. Model A - Full statistics of all direct and indirect relationships.

		Standardized estimate	*p*-value	95% CI
**Direct effects**				
LR1				
	Age (in year 2015)	0.022	0.175	(-0.010, 0.054)
	Sex			
	Male	*Ref*		
	Female	**-0.524**	< 0.001	(-0.544, -0.504)
	Ethnicity			
	Javanese	*Ref*		
	Sundanese	**-0.037**	< 0.050	(-0.063, -0.012)
	Others	-0.009	0.513	(-0.037, 0.019)
	Highest education level			
	No education	*Ref*		
	Elementary	-0.047	0.097	(-0.103, 0.009)
	High school	**-0.117**	< 0.001	(-0.179, -0.054)
	College/University	**-0.165**	< 0.001	(-0.211, -0.120)
LR2				
	Age (in year 2015)	0.036	0.056	(-0.001, 0.072)
	Sex			
	Male	*Ref*		
	Female	**-0.074**	< 0.001	(-0.103, -0.044)
	Ethnicity			
	Javanese	*Ref*		
	Sundanese	**0.084**	< 0.001	(0.055, 0.113)
	Others	**-0.127**	< 0.001	(-0.158, -0.095)
	Highest education level			
	No education	*Ref*		
	Elementary	**0.172**	< 0.001	(0.106, 0.238)
	High school	**0.330**	< 0.001	(0.257, 0.403)
	College/University	**0.319**	< 0.001	(0.267, 0.372)
Physiological Load mediator				
	LR1	**-0.053**	< 0.050	(-0.088, -0.019)
	LR2	**0.045**	< 0.050	(0.014, 0.076)
	Age (in year 2015)	**0.251**	< 0.001	(0.218, 0.285)
	Sex			
	Male	*Ref*		
	Female	**0.106**	< 0.001	(0.071, 0.142)
	Ethnicity			
	Javanese	*Ref*		
	Sundanese	-0.004	0.785	(-0.036, 0.027)
	Others	-0.023	0.148	(-0.055, 0.008)
	Highest education level			
	No education	*Ref*		
	Elementary	0.060	0.087	(-0.009, 0.128)
	High school	0.060	0.120	(-0.016, 0.135)
	College/University	**0.059**	< 0.050	(0.002, 0.116)
HbA1c				
	LR1	-0.012	0.461	(-0.045, 0.020)
	LR2	**0.033**	< 0.050	(0.002, 0.064)
	Physiological Load mediator	**0.207**	< 0.001	(0.171, 0.244)

		Standardized estimate	*p*-value	95% CI
	Age (in year 2015)	**0.144**	< 0.001	(0.108, 0.180)
	Sex			
	Male	*Ref*		
	Female	**-0.071**	< 0.001	(-0.107, -0.035)
	Ethnicity			
	Javanese	*Ref*		
	Sundanese	-0.003	0.875	(-0.034, 0.029)
	Others	0.018	0.276	(-0.014, 0.049)
	Highest education level			
	No education	*Ref*		
	Elementary	-0.047	0.219	(-0.121, 0.028)
	High school	-0.022	0.604	(-0.107, 0.062)
	College/University	-0.027	0.399	(-0.089, 0.035)
Intercepts	LR1	**0.661**	< 0.001	(0.485, 0.837)
	LR2	**-0.508**	< 0.001	(-0.712, -0.304)
	Physiological Load mediator	0.207	0.354	(-0.291, 0.104)
	HbA1c	**5.447**	< 0.001	(5.039, 5.855)
Residual variances	LR1	**0.709**	< 0.001	(0.688, 0.731)
	LR2	**0.913**	< 0.001	(0.896, 0.930)
	Physiological Load mediator	**0.920**	< 0.001	(0.904, 0.936)
	HbA1c	**0.923**	< 0.001	(0.905, 0.941)
**Indirect effects on HbA1c via Physiological Load mediator**				
HbA1c				
	LR1	**-0.011**	< 0.050	(-0.019, -0.004)
	LR2	**0.009**	< 0.050	(0.003, 0.016)
R^2	LR1	0.291		
	LR2	0.087		
	Physiological Load mediator	0.080		
	HbA1c	0.077		
Model fit indices	RMSEA	0.014		
	CFI	1.000		
	TLI	0.989		
	SRMR	0.002		

Significant estimates at p < 0.05 are shown in bold. All values were rounded off to 3 decimal places.
